# Rhegmatogenous Retinal Detachment Surgery in Elderly People over 70 Years Old: Visual Acuity, Quality of Life, and Cost-Utility Values

**DOI:** 10.1371/journal.pone.0110256

**Published:** 2014-10-17

**Authors:** Yingyan Ma, Xiaohua Ying, Haidong Zou, Xiaocheng Xu, Haiyun Liu, Lin Bai, Xun Xu, Xi Zhang

**Affiliations:** 1 Department of Ophthalmology, Shanghai First People’s Hospital, Shanghai Jiao Tong University, Shanghai, China; 2 School of Public Health, Fudan University, Shanghai, China; 3 Shanghai Eye Disease Prevention & Treatment Center, Shanghai, China; Sun Yat-sen University, China

## Abstract

**Background and Purpose:**

To evaluate the influence of rhegmatogenous retinal detachment (RRD) surgery on elderly patients in terms of visual acuity, vision-related quality of life and its cost-effectiveness.

**Methods:**

Elderly patients over 70 years old, who were diagnosed and underwent RRD surgery at Shanghai First People's Hospital, Shanghai Jiao Tong University, China, from January 1, 2009, through January 1, 2013. The participants received scleral buckling surgery and vitreous surgery with or without scleral buckling under retrobulbar anesthesia. We followed the patients for 1 year and collected best-corrected visual acuity (BCVA), vision-related quality of life, and direct medical costs data. Utility values elicited by time-trade-off were analyzed to determine the quality of life. Quality-adjusted life years (QALYs) gained in life expectancy were calculated and discounted at 3% annually. Costs per QALY gained were reported using the bootstrap method. Further analyses were made for two age groups, age 70–79 and age over 80 years. Sensitivity analyses were performed to test stability of the results.

**Results:**

98 patients were included in the study. The BCVA significantly improved by 0.53±0.44 (Logarithm of the Minimum Angle of Resolution (logMAR)) at the 1-year postoperative time point (p<0.001). Utility values increased from 0.77 to 0.84 (p<0.001), and an average of 0.4 QALYs were gained in the life expectancy. Costs per QALY gained from the RRD surgery were 33,186 Chinese Yuan (CNY) (5,276 US dollars (USD))/QALY; 24,535 CNY (3,901 USD)/QALY for the age group of 70–79 years and 71,240 CNY (11,326 USD)/QALY for the age group over 80 years.

**Conclusions:**

RRD surgery improved the visual acuity and quality of life in the elderly patients over 70 years old. According to the World Health Organization’s recommendation, at a threshold of willingness to pay of 115,062 CNY (18,293 USD)/QALY, RRD surgery is cost effective in the elderly patients.

## Introduction

Rhegmatogenous retinal detachment (RRD) is the most common type of retinal detachment, and it severely threatens visual acuity and vision-related quality of life [1]–[3]. Proper and timely treatments, such as scleral buckling surgery and vitreous surgery, can largely restore visual acuity and permit a certain degree of improvement in vision-related quality of life [4], [5]. However, in most developing countries with limited medical resources, the treatment for retinal detachment has been a low priority [6]. Despite the effectiveness of RRD surgery, the costs are not small. Brown and associates reported the costs for vitreoretinal surgery to be between 7,109 US dollars (USD) and 9,607 USD in patients with severe proliferative vitreoretinopathy [7]. Two recent studies performed in America calculated the costs for the two types of surgery varying from 4,048 USD to 7,940 USD [8], [9]. In our previous study, an average cost of 11,384 Chinese Yuan (CNY) (1,810 USD, 1 USD = 6.29 CNY, 2012.12.31) was determined for RRD surgery in Shanghai [10]. Although comparatively lower than the cost reported in America, it is still a large burden for most families in China.

The annual incidence of RRD ranged from 6.3 to 17.9 per 100,000 population globally [11]. Age is one risk factor for RRD [11]–[13]. In elderly people who are 70 to 79 years old, the incidences vary from 15.21 to 50 per 100,000 worldwide [11]. In a recent study conducted in the Netherlands, the annual incidence of RRD was reported to be 21.43 per 100,000 for people aged 85–89 years old [14]. However, age was also a negative predictor for optimal visual outcomes after RRD surgery. As demonstrated in many previous studies, patients of older age have inferior functional results, namely best-corrected visual acuity, than younger patients [15]–[19]. Therefore, in clinical practice, it is not unusual that many elderly people give up on the surgery considering the expensive medical costs, the uncertain visual outcomes, and the relatively short remaining life years. These elderly people will definitely experience severe visual impairment, and as a result, losses of quality of life. As shown in a survey of a community population aged over 60 years in Shanghai, retinal detachment was the fifth leading cause of blindness [20].

Cost-utility analysis is one method of economic evaluation that incorporates the utility value in the form of quality-adjusted life years (QALYs) with costs to calculate how much money should be spent on each QALY gained for certain medical interventions [21], [22]. One advantage of cost-utility analysis is that it allows comparison among different disciplines by a common unit of measure (costs/QALY). Therefore, policymakers can identify relative priorities when determining resource allocation among medical interventions [21], [22]. In Brown’s study, the costs/QALY were between 40,252 USD/QALY and 62,383 USD/QALY for vitreous surgery in patients complicated with severe proliferative vitreoretinopathy [7]. In the recent American study, the costs/QALY varied from 1,377 USD/QALY to 2,243 USD/QALY for scleral buckling and vitreous surgery [9]. In Shanghai, the costs/QALY were 13,794 CNY (2,193 USD)/QALY for normal RRD patients throughout their life expectancies [10]. However, the cost-effectiveness of RRD surgery for elderly patients has not been evaluated in the existing literatures.

As we enter an aging society, along with the popularity of cataract surgery and high rates of myopia, we anticipate a greater number of elderly patients suffering from RRD [11]. Therefore, it is worthwhile to explore how much the elderly people could benefit from RRD surgery and whether performing RRD surgery is cost-effective in this population. This study presented the outcomes of RRD surgery in terms of visual acuity and vision-related quality of life and calculated its cost-effectiveness in an elderly population.

## Methods

### Ethics

All participants gave their written informed consent. The study was conducted according to the tenets of the Declaration of Helsinki and was approved by the Institutional Review Board at Shanghai First People's Hospital, Shanghai Jiao Tong University.

### Participants and investigations

Between January 1, 2009, and January 1, 2013, study participants included elderly patients over the age of 70 years who were newly diagnosed with RRD (in at least one eye) and subsequently underwent surgery in the Shanghai First People's Hospital, Shanghai Jiao Tong University. Patients were excluded if they had other severe eye diseases, physical disability or mental disorders.

Participants were interviewed before and one year after surgery. Data were collected for age, gender, educational level, systemic diseases, duration of time from RRD symptoms to RRD surgery (or, if no symptoms, time since diagnosis), range of detachment in quadrants, macular-on or macular-off, RRD surgery type, surgical procedures, best-corrected visual acuity (BCVA), costs, utility value, and complications of surgery. BCVA was also collected at the 3-month postoperative time point. Surgical procedures were classified as scleral buckling surgery and vitreous surgery with or without scleral buckling. A skilled interviewer (HDZ), who did not take part in any of the examinations or follow-up with the participants, administered the questionnaire and provided assistance when required.

### Utility values and costs

Utility values were obtained by using the time trade-off (TTO) method at pre-operative and 1-year post-operative time points. Participants were asked how many additional years they expected to live and how many of these theoretical remaining years they would be willing to give up in return for guaranteed, permanently perfect vision. The utility value was then calculated by subtracting the quotient of these numbers (years given up/years to live) from 1.0 [23]. For example, if a 70-year-old RRD patient with a self-perceived life expectancy of 10 additional years would like to trade 2 years to get rid of his or her visual impairment caused by RRD, the utility value related to his or her RRD would be (1.0–2/10) = 0.8.

Costs were calculated from the patient perspective, and all of these costs were inflated to 2012 values by utilizing the consumer price index for health care [24]. Self-designed investigation forms were distributed to record the medical costs associated with RRD surgery for the first diagnosed eye, including costs of pharmaceuticals, examinations, treatment, anesthesia, surgery, hospitalization, and transportation fees. Downstream costs induced by surgery were also collected during the 1-year follow up, such as the costs for the removal of silicone oil and treatment for complications including cataract surgery, treatment of corneal edema, and intraocular hypertension. If the second eye also received RRD surgery during the 1-year study period, costs incurred by this surgery were also recorded. The costs collected in this research were associated with the Chinese medical insurance unified prices; hence, they were representative of the medical costs charged by other hospitals in Shanghai and in other cities in China. Only direct costs were included. Indirect costs, such as loss of productivity and leisure time, were not incorporated into this investigation.

### Model design

According to our previous research [25], we assumed that the utility value in RRD patients increased steadily in the first year and then remained stable. Hypothetical patients designed as no-treatment controls were expected to obtain the same pre-operative utility values as the participants. Additionally, it was assumed that their utility values remained constant and that no medical costs related to RRD treatment had ever been incurred in our hypothetical patients.

Because the benefits of RRD surgery are life-long, we carried out a cost-utility analysis over the remaining life expectancy for RRD surgery. Lacking evidence of utility change over the remaining lifetime of RRD patients, and in accord with other economic evaluations within ophthalmology [7], [26], [27], we assumed that the vision-related quality of life would remain constant over the life expectancy. The age and gender-specific life expectancy table from the Health Profile China was used to calculate life expectancy QALYs gained [28]. In the control group, hypothetical patients who did not receive surgery would also remain at their preoperative health status throughout their life expectancies with no medical expenditures related to RRD. The QALYs were discounted at 3% annually in the baseline analysis. Because all medical costs were paid over a 1-year period, they were not discounted. The incremental cost-effectiveness ratio (ICER) was then calculated to evaluate the life-span cost-utility result of RRD surgery, compared with the no-treatment option.

Moreover, we performed subgroup analyses by dividing the participants into two age groups: 70–79-year-olds and over 80-year-olds. Costs, QALYs gained, and the ICER were calculated for each age group to explore the cost-effectiveness of RRD surgery in different age intervals. Additional analyses for scleral buckling surgery and vitreous surgery were also calculated.

### Threshold ratio of willingness to pay

In China, there is no official guiding document or consensus on the threshold ratio of willingness to pay (WTP); hence, we adopted the standard posed by the World Health Organization (WHO). As recommended by the WHO, if the ICER is less than three times the gross domestic product (GDP) per capita, it can be regarded as cost-effective, and if the ICER is less than the GDP per capita, the intervention can be regarded as highly cost-effective [29]. This approach has already been utilized in several economic evaluations in China [30], [31]. With a 2012 GDP per capita in China of 38,354 CNY, we used 115,062 CNY (18,293 USD)/QALY as the threshold ratio of willingness to pay to determine whether the RRD surgery was cost-effective in the elderly population.

### Statistical analysis

Costs and QALYs are usually highly skewed, and the uncertainty of the ICER is difficult to present using traditional statistical methods; therefore, we performed a bootstrapping with 1,000 replications to calculate mean costs, mean QALYs, the ICERs and the uncertainty of those results [32]–[34]. The percentile method (2.5 and 97.5 percentiles from the bootstrapped sample) was used to estimate 95% confidence intervals (CIs) for the incremental costs and QALYs [21], [33]. Furthermore, cost-effectiveness acceptability curves (CEACs) were drawn, representing the probability of RRD surgery being more cost-effective than no treatment for different thresholds of willingness to pay per QALY [35], [36]. The Mann-Whitney U test was used to compare the differences of costs, utility values, QALYs, and duration time of RRD between the two age groups for the non-normal distribution of the variables. An independent sample t-test was used for continual data of normal distribution, and the Pearson chi-square test was used for the categorical data. The paired sample t-test was used for testing differences between BCVA before and after surgery, and the Wilcoxon signed rank test was used for differences between utility values.

A value of P<0.05 was regarded as statistically significant. All bootstrap analyses were performed using Microsoft Excel 2007 software, and other analyses were calculated by SPSS 16.0.

### Sensitivity analyses

We conducted sensitivity analyses to test the robustness of the results. First, we tested the impact of varying the discount rate from the 3% assumed in the baseline scenario to between 0% and 5% for lifespan analyses, as recommended [21]. Second, we floated the costs and QALYs to 10% individually and simultaneously. Subsequently, we calculated the upper limits for the extent of the increasing costs, or the decreasing QALYs, to overturn the final determination. Finally, we performed an additional analysis by excluding patients who had undergone bilateral RRD surgery, which might have had a potential influence on the results [7].

## Results

A total of 98 patients fulfilled study inclusion criteria, with 57 patients aged 71 to 79 years old and 41 patients aged 81 to 87 years old. Basic socio-demographic and clinical data for the patients appear in Table 1. There was no significant difference between the two age groups in terms of basic characteristics, including type of surgery. During the 1-year follow up, no severe systemic diseases occurred in any participant.

**Table 1:** Characteristics and surgical data for 98 elderly RRD patients.

	Total	70s	80s	P value*
**No. of patients**	98	57	41	
**Average age [Mean (SD)]**	78.49(4.36)	75.33(2.48)	82.88(1.90)	<0.001
**Male [No. (%)]**	54(55.1)	35(61.40)	19(46.34)	0.139
**Education time >10 years [No. (%)]**	50(51.02)	32(56.14)	18(43.90)	0.232
**Duration of symptoms [Weeks, Median (Range)]**	5[1]–[27]	6[1]–[23]	4[1]–[27]	0.550
**More than 2 quadrants detached [No. (%)]**	78(79.60)	46(80.70)	32(78.05)	0.748
**Macular-off [No. (%)]**	68(69.39)	40(70.18)	28(68.29)	0.842
**Types of surgery**				0.191
**Scleral buckling surgery [No. (%)]**	31(31.63)	21(36.84)	10(24.39)	
**Vitreous surgery [No. (%)]**	67(68.37)	36(63.16)	31(75.61)	
**Bilateral surgery rate [No. (%)]**	13(13.27)	9(15.79)	4(9.76)	0.385
**Complication rate [No. (%)]**	66(67.35)	35(61.40)	31(75.61)	0.139

*A comparison was made between age groups of patients in their 70s and 80s. An independent sample t-test was used for continual data of normal distribution, a Mann Whitney U test for non-normal distribution data, and the Pearson chi-square test for the categorical data. A value of P<0.05 was regarded as statistically significant.

RRD, rhegmatogenous retinal detachment; SD, standard deviation.

The best-corrected visual acuity significantly improved at the 3-month post-operative time point compared with the preoperative time (paired sample t-test, p<0.001 for overall participants, patients in their 70s, and patients in their 80s). At the 1-year post-operative time point, BCVA also improved significantly (paired sample t-test, p<0.001 for overall participants, patients in their 70s, and patients in their 80s) (Table 2). The utility values significantly increased 1 year after the surgery (Wilcoxon signed rank test, p<0.001 for the whole group, patients in their 70s, and patients in their 80s). The results of the baseline cost-utility analysis for the two age groups are shown in Table 3.

**Table 2:** Outcomes of visual acuity for RRD surgery in an elderly population (n = 98).

	Total	70s	80s	P value*
**BCVA before surgery [LogMAR, Mean (SD)]**	1.06(0.50)	1.05(0.50)	1.09(0.52)	0.682
**BCVA 3 months after surgery [LogMAR, Mean (SD)]**	0.74(0.35)	0.71(0.34)	0.78(0.36)	0.334
**Difference of BCVA during the 3 months [LogMAR, Mean (SD)]**	0.32(0.35)	0.34(0.36)	0.31(0.34)	0.709
**BCVA 1 year after surgery [LogMAR, Mean (SD)]**	0.54(0.26)	0.53(0.25)	0.55(0.29)	0.650
**Difference of BCVA during the 1 year [LogMAR, Mean (SD)]**	0.53(0.44)	0.52(0.40)	0.54(0.49)	0.845

*A comparison was made between age groups of patients who were in their 70s and 80s using independent samples t-tests. A value of P<0.05 was regarded as statistically significant.

RRD, rhegmatogenous retinal detachment; BCVA, best-corrected visual acuity; LogMAR, Logarithm of the Minimum Angle of Resolution; SD, standard deviation.

**Table 3:** Results of the baseline cost-utility analysis for RRD surgery in an elderly population (bootstrap, n = 1000).

	Total	70s	80s	P value*
**Utility value before surgery** **[Mean (SD)]**	0.77(0.12)	0.76(0.13)	0.79(0.11)	0.131
**Utility value 1-year after surgery** **[Mean (SD)]**	0.84(0.08)	0.84(0.07)	0.84(0.09)	0.352
**Difference of utility values during** **the 1 year [Mean (SD)]**	0.07(0.07)	0.08(0.08)	0.05(0.06)	0.096
**QALYs gained in life-expectancy** **[Mean (95% CI)]**	0.40(0.31–0.50)	0.55(0.41–0.72)	0.18(0.12–0.24)	
**Mean Costs [CNY(USD)]**	12,992(2,066)	13,319(2,117)	12,484(1,985)	
**Costs 95% CI [CNY(USD)]**	11,984(1,905)−14,088(2,239)	11,818(1,879)−14,800(2,353)	11,131(1,769)−13,910(2,211)	
**Mean ICER [CNY(USD)/QALY]**	33,186(5,276)	24,536(3,901)	71,240(11,326)	

*Comparisons were made between age groups of patients in their 70s and 80s using a Mann Whitney U test. A value of P<0.05 was regarded as statistically significant.

RRD, rhegmatogenous retinal detachment; CNY, Chinese Yuan; USD, US dollar; SD, standard deviation; QALY, quality-adjusted life year; CI, confidence interval; ICER, incremental cost-effectiveness ratio.

Over their remaining life expectancies, participants could achieve an average of 0.4 QALYs by having the retinal detachment surgery. For patients in their 70s, the QALYs gained were more than three times greater than for those of the patients in their 80s. According to the threshold willingness to pay, which was 115,062 CNY (18,293 USD)/QALY in 2012, the retinal detachment surgery is cost-effective for all elderly patients. The ICER for patients in their 80s is approximately three times higher than that of those in their 70s, though it is still a cost-effective intervention.

**Figure 1:**
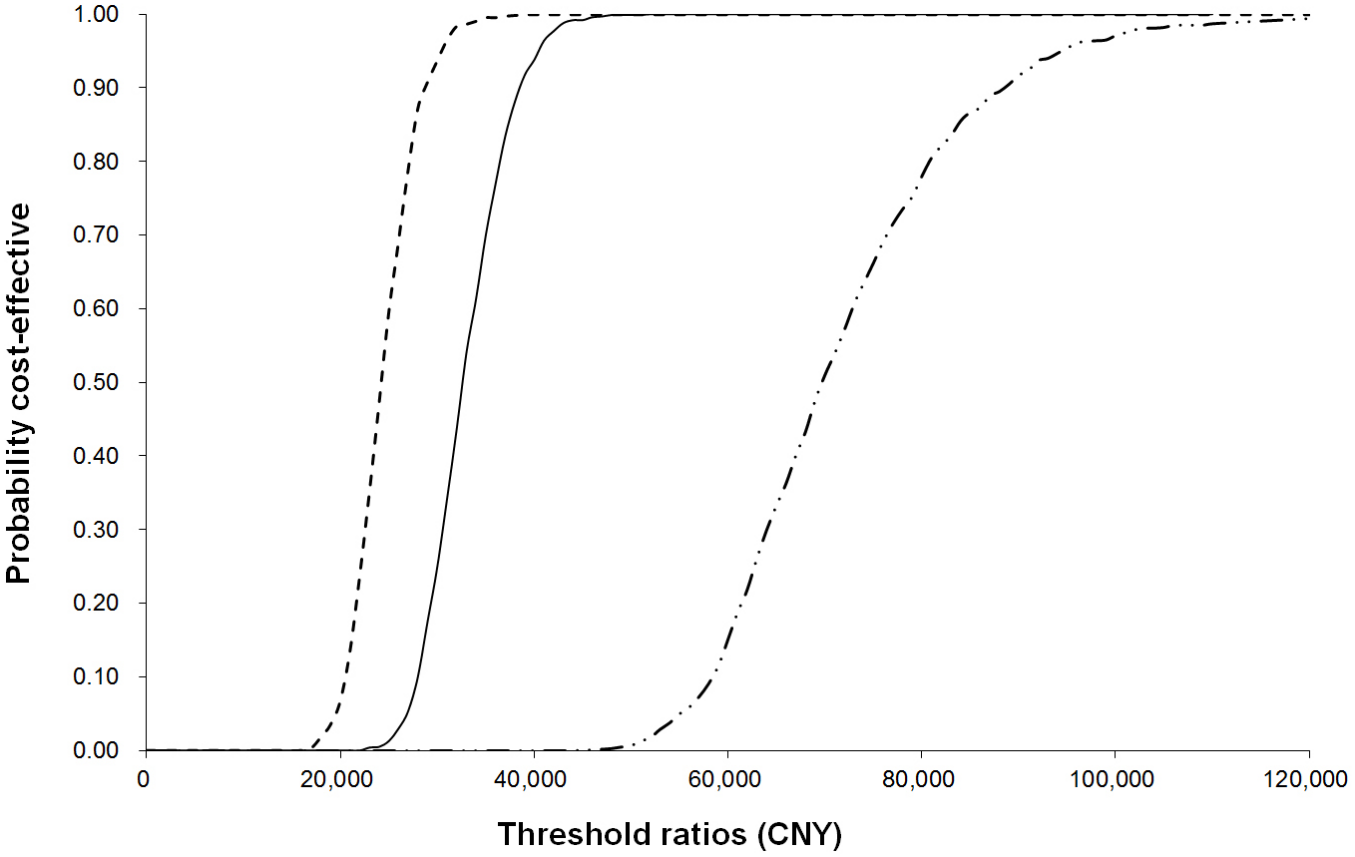
CEACs of life expectancy analysis for RRD surgery in the 70–79-year-olds, the above-80-year-olds, and all the elderly patients. The upper most dashed line represents the CEAC for RRD surgery in the 70–79-year-olds. The solid line represents the CEAC for RRD surgery in all of the elderly people. The dot-dashed line represents the CEAC for RRD surgery in the above-80-year-olds. RRD, Rhegmatogenous retinal detachment; CNY, Chinese yuan; QALY, quality-adjusted life year; CEAC, Cost-effectiveness acceptability curve.

With a WTP of 115,062 CNY (18,293 USD) per QALY, there is a 100%, 100%, and 99.2% chance of RRD surgery being cost-effective for all of the elderly, patients in their 70s, and patients in their 80s, respectively. The CEAC (Figure 1) of the over-age-80 group is lower than that of the 70–79-year-old group, which indicates that RRD surgery is more cost-effective for those in their 70s than in their 80s. In the absence of an officially recognized threshold WTP, we further listed the threshold ratios of WTP for the surgery with cost-effective chances of 50%, 70%, and 90% (Table 4).

**Table 4:** Threshold ratios of WTP (CNY (USD)/QALY) for RRD surgery to be cost-effective at probabilities of 50%, 70% and 90% compared with no treatment option.

	WTP [CNY(USD)/QALY]		
Probability of being cost-effective	All (n = 98)	70s (n = 57)	80s (n = 41)
**50%**	33,000(5,246)	24,000(3,816)	70,000(11,129)
**70%**	35,000(5,564)	26,000(4,134)	76,000(12,083)
**90%**	39,000(6,200)	29,000(4,610)	88,000(13,991)

WTP, willingness to pay; CNY, Chinese Yuan; USD, US dollar; QALY, quality-adjusted life year.

The results of additional analyses for the two major types of surgery were presented in table 5. The vitreous surgery was a little more expensive than the scleral buckling surgery, however obtained more QALYs for the elderly patients as well; hence, the costs per QALYs for vitreous surgery were less expensive than for scleral buckling surgery in the elderly population.

**Table 5:** Results of cost-utility analysis for the two surgery types in an elderly RRD population (bootstrap, n = 1000).

	Scleral buckling surgery (n = 31)	Vitreous surgery (n = 67)
**Mean Costs [CNY(USD)]**	12,804(2,036)	13,027(2,071)
**Costs 95% CI [CNY(USD)]**	10,813(1,719)−14,737(2,343)	11,698(1,859)−14,343(2,281)
**QALYs gained in** **life-expectancy [Mean (95% CI)]**	0.36(0.24–0.50)	0.41(0.29–0.56)
**Mean ICER [CNY(USD)/QALY]**	36,513(5,805)	32,287(5,133)

RRD, rhegmatogenous retinal detachment; CNY, Chinese Yuan; USD, US dollar; QALY, quality-adjusted life year; ICER, incremental cost-effectiveness ratio.

Table 6 presents the results of the sensitivity analyses. In general, the sensitivity analyses do not influence the decisions based on the results of the baseline analysis. Altering the discount rate from 3% in the baseline scenario to 0% decreases the ICERs; however, using a 5% discount rate increases the ICERs. Raising the costs by 10% and reducing the QALYs gained by 10% mildly increases the ICERs. With changes in both the costs and the QALYs, the ICERs still did not exceed the threshold of 115,062 CNY (18,293 USD)/QALY. To overturn the cost-effectiveness of the RRD surgery, the costs have to be 2.5 times more than the existing costs and the QALYs gained should be decreased by 75% from the baseline. Excluding bilateral RRD patients enhances the ICERs and makes RRD surgery less cost-effective for all of the elderly patients and in each age group.

**Table 6:** A summary of ICERs (CNY (USD)/QALY) in sensitivity analyses for RRD surgery in an elderly population (n = 98).

	All	70s	80s
**0% discount**	30,142(4,792)	22,072(3,509)	69,756(10,091)
**5% discount**	35,092(5,579)	25,933(4,123)	73,961(11,758)
**Cost+10%**	36,111(5,740)	26,849(4,269)	78,790(12,526)
**QALYs−10%**	37,049(5,891)	27,160(4,318)	80,863(12,856)
**Costs+10% and QALYs−10%**	40,607(6,456)	29,743(4,729)	89,210(14,183)
**Costs limit**	+250%	+365%	+68%
**QALYS limit**	−75%	−81%	−42%
**Excluding bilateral RRD patients**	38,243(6,080)	25,825(4,106)	72,250(11,486)

ICER, incremental cost-effectiveness ratio; CNY, Chinese Yuan; USD, US dollar; RRD, rhegmatogenous retinal detachment; QALY, quality-adjusted life year.

## Discussion

This article is the first to evaluate the visual outcomes and health economic value of RRD surgery in the elderly population. RRD surgery improved the visual acuity and quality of life for the elderly patients and was cost-effective in consideration of their life expectancies, with ICERs ranging from 30,142 CNY (4,792 USD)/QALY to 40,607 CNY (6,456 USD)/QALY. For patients above 80 years of age, although the ICER was about three times higher than that for patients aged 70–79 years, the RRD surgery remained a cost-effective intervention.

Compared with other studies, the patients in this study are relatively older, as most studies included patients with an average age of approximately 60 years old [37]–[39]. Additionally, the duration of symptoms until surgery is longer, although it is similar to those reported in developing countries [40], [41]. In China, 60–70% of RRD patients reside in remote areas or small villages and lack knowledge about RRD [42]; thus, the elderly patients received treatment after a longer time period from their diagnoses, which resulted in relatively poorer preoperative visual acuity and higher rates of macular-off compared with participants in other studies.

However, the surgery brought significant improvement to the visual acuity of these patients. The BCVA of the elderly patients increased continuously from 3 months to 1 year postoperatively in the study. The average improvement in BCVA was 0.53±0.44 at the 1-year postoperative time point for all of the participants. Although age was reported to be a negative predictor of visual acuity outcomes after RRD surgery in many publications [15]–[19], the increase in BCVA in our study was no less than that reported in other studies that also included younger patients whose average age was approximately 60 years old [25], [37], [38], [43]. Similarly, in the SPR study, no relationship was observed between age and inferior visual outcomes [44]. Due to the inconsistency of the baseline status of RRD between the different study groups, the results in our study cannot be directly compared with other studies and we are therefore not be able to conclude whether age is a negative predictor for visual outcome. In any case, the significant improvement in BCVA indicates that RRD surgery is beneficial for patients over 80 years old as well as patients 70–79 years old.

In recent years, increasing emphasis has been put on evaluating the vision-related quality of life in ocular diseases. To reflect how the retinal detachment surgery influences the patients’ quality of life, we investigated preference-based utility values before and 1 year after the surgery among the elderly patients. Utility analysis, unlike some other quality of life measurements, enables comparisons of quality of life across different health statuses in medicine and further analyses of cost-utility results for resource allocations [45]. The patients reported statistically significant increases in utility values postoperatively, rising from an average of 0.77 to 0.84. The patients above 80 years old seem to benefit less than those in the 70 to 79 years old group do, as demonstrated by the 1-year postoperative utility value; however, the ambiguous p value (0.096) makes it difficult to determine whether this difference is meaningful. Moreover, the change in utility values in the elderly population is similar to what we investigated in a younger population with an average age of 54 years old (P = 0.427) [4]. A summary of comparisons with our previous study is presented in Table 7.

**Table 7:** Comparisons of surgical outcomes between the normal RRD patients and the elderly patients [4], [10].

	Normal population	Elderly population	P value*
**No. of Patients**	117	98	
**Age [Mean (SD)]**	54.28(10.30)	78.49(4.36)	<0.001
**Type of surgery**			<0.001
**Scleral buckling surgery [No. (%)]**	68(58.12)	31(31.63)	
**Vitreous surgery [No. (%)]**	49(41.89)	67(68.37)	
**Preoperative BCVA**	1.06(0.63)	1.06(0.50)	0.832
**BCVA after 3 months**	0.68(0.40)	0.74(0.35)	0.227
**BCVA after 1 year**	0.51(0.32)	0.54(0.26)	0.456
**Difference of BCVA in 3 months**	0.38(0.40)	0.32(0.35)	0.314
**Difference of BCVA in 1 year**	0.55(0.48)	0.53(0.44)	0.773
**Utility value before surgery**	0.77(0.12)	0.77(0.12)	0.806
**Utility value after 1 year**	0.83(0.10)	0.84(0.08)	0.610
**Difference of utility value in 1 year**	0.06(0.09)	0.07(0.07)	0.427
**Costs [Bootstrap 1000 times;** **Mean (95% CI); CNY]**	11,384(10,338–12,563)	12,992(11,984–14,088)	
**QALYs gained in life time [Bootstrap 1000 times;** **Mean (95% CI)]**	0.88(0.64–1.13)	0.40(0.31–0.50)	
**ICERs [Bootstrap 1000 times;** **Mean; CNY/QALY]**	13,794	33,186	

*An independent sample t-test was used for continual data of normal distribution, a Mann Whitney U test for non-normal distribution data, and the Pearson chi-square test for the categorical data. A value of P<0.05 was regarded as statistically significant.

RRD, rhegmatogenous retinal detachment; SD, standard deviation; BCVA, best -corrected visual acuity; CI, confidence interval; CNY, Chinese Yuan; QALY, quality-adjusted life year.

When the benefit of lifetime was considered, the QALYs gained in patients aged 70–79 years old were approximately three times greater than in patients over 80 years old. Compared with our previous study [10], the QALYs gained in the elderly population were relatively small: 0.40 QALYs for the elderly compared to 0.88 QALYs for an average group of patients (Table 7). This is likely because older people have theoretically fewer remaining life years than younger people do. Consequently, the ICER, which combined the costs and QALYs gained, was higher in the elderly population compared to an average group of patients. The ICER in patients over 80 years old was three times higher than that in patients in their 70s. Despite the lower cost-effectiveness of RRD surgery in the elderly patients, the treatment was still below the threshold of willingness to pay of China, even for patients over 80 years old.

In the subgroup analyses for the two types of surgery, the vitreous surgery was less expensive for each QALYs gained than scleral buckling surgery in the elderly population, however, the discrepancy was not large. Unlike what we observed in the normal patients [10], the elderly people seemed to benefit less from scleral buckling surgery, probably because that the elderly patients usually have more complicated and diverse conditions than the normal patients do, which enables the comparable costs for the two types surgery. This might also be the reason that the proportion of vitreous surgery was higher in the elderly population than in the normal patients (Table 7), since surgeons’ preference of choosing the vitreous surgery for complicated situation.

Elderly patients do not always accept surgery for RRD. On the one hand, the costs of RRD surgery are still relatively high for most people in our country, despite the fact that the National Health Insurance policy might cover some of the expenses [46]. On the other hand, elderly people might have fewer demands for vision-related quality of life because daily life activities are reduced and their life expectancy is relatively short. Additionally, lacking knowledge about health, they might be anxious about the disadvantages of the surgery, such as discomfort, complications, or risk of death. As a result, quite a few elderly people ultimately choose not to undertake the surgical option, which is the reason for the relatively small sample size in this population. It is difficult to persuade such patients to have surgery, yet it is considered unethical for an ophthalmologist to allow these patients to return home without treatment. Our study provides evidence that RRD surgery can improve visual acuity and the quality of life, and it is a cost-effective intervention in people over 70 years old. Therefore, ophthalmologists should persuade such elderly RRD patients to have surgery. In the interim, this health economic evaluation of RRD surgery may also provide a valuable tool for governmental agencies in formulating the National Health Insurance policy.

There are some limitations of this study. First, the study lacks real patient controls; however, not offering treatment for RRD patients violates the tenets of the Declaration of Helsinki, preventing approval by an ethical committee. Secondly, the study period was not long enough to truly reflect the long-term cost-utility of RRD surgery. The results of the long-term analysis were based on the assumption that no more costs associated with RRD surgery would be incurred and that patients would retain their utility value of the 1-year postoperative time point throughout their remaining life years. In the real world, outpatient service fees and transportation fees should be included as well, even if the costs are trivial compared with the surgery expenditures during the first year. Additionally, the utility value would deteriorate both in the treatment group and in the control group over the course of the life expectancy; however, some of the declining factors might counteract each other, making their influence unclear. Third, the study population was relatively small, and all the participants were from one teaching hospital. The results might be more presentative for large cities with similar economic conditions as Shanghai than for small towns in China. In future work, studies with more participants from different regions and longer follow-up times are desirable to facilitate cost-utility analyses that are more comprehensive. Finally, we did not include indirect costs, and this exclusion would probably underestimate the economic value of the surgery, as people in the control group had severe visual impairment, which might influence their living independency and incur expenses for nursing.

To conclude, the RRD surgery greatly improves visual acuity and the quality of life in elderly people in China. Compared to other studies that include younger populations, the visual acuity and vision-related quality of life outcomes were similar. Considering the shorter life expectancy, the elderly patients obtain fewer QALYs from RRD surgery, and thus, a higher cost per QALY was found. However, it is still cost-effective for elderly people to have RRD surgery, even for patients over 80 years old.
